# Family capital, social stratification, and access to higher education: An empirical study in mainland China

**DOI:** 10.3389/fpsyg.2022.1035715

**Published:** 2023-01-10

**Authors:** Qinyi Tan, Chencheng Li, Pei Wu, Safdar Abbas, Luyan Teng

**Affiliations:** ^1^Center for Studies of Education and Psychology of Ethnic Minorities in Southwest China, Southwest University, Chongqing, China; ^2^China School of Foreign Languages and Literature, Southwest University, Chongqing, China; ^3^College of International Education, Sichuan International Studies University, Chongqing, China

**Keywords:** Bourdieu’s capital theory, China family panel studies, blue-collar stratum, cultural capital, higher education, logistic regression model

## Abstract

This paper employs Bourdieu’s theory of capital—focusing on family cultural, social, and economic capital—to research the early-stage mechanism through which access to higher education is formed. While all three types of capital play a significant role in acquiring higher education, most studies tend to focus on just one type of capital. In recent years, domestic scholars have also analyzed in detail the family factors affecting children’s access to higher education (CAHE); however, they have not yet explained the mechanism by which these factors influence CAHE, and authentic tests are rare. Therefore, based on existing research, this paper uses the theoretical concept of family capital to reveal how contemporary Chinese families affect their CAHE. This paper analyzes the relationship between family capital, social stratification, and access to higher education opportunities using an econometric model based on baseline data from the China Family Panel Studies (CFPS) from 2010 to 2020, with 10,318 participants, including 4,419 females and 5,899 males. The results of a binary logistic regression analysis show that the possession of family cultural and economic capital has a direct positive influence on CAHE. Children from the elite stratum often benefit more from the accumulation of family cultural capital. Moreover, although it does not form a distinct stratum, the possession of family social capital also significantly influences children’s access to higher education. Driven by China’s political, economic, and social environment, some children from the blue-collar stratum have a comparative advantage in terms of access to higher education. The possession of family capital is an important factor in the stratification of CAHE, and cultural capital is the most influential type of capital. Parents with a low level of education should be encouraged to become engaged in schools and communities to take professional courses in assisted learning, emotional counseling, decision-making, and voluntary service.

## 1. Introduction

Educational equity is related to national development and people’s livelihoods (Duerrenberger and Warning, 2018). Against a backdrop of educational reform, theoretical research relating to educational equity provides a valuable assessment of change. Higher education is becoming increasingly integrated into our lives as an important tool to help improve the status of children from social strata that possess few social resources (Li and Qiu, 2018). Academic qualifications and access to social resources are strongly correlated (Zhao and Qin, 2021). People with higher academic qualifications can achieve higher prestige occupations and obtain more income and rights (de Wit and Altbach, 2021).

Among the many factors affecting access to higher education opportunities is a family’s amount of capital, which has a direct differential influence on the acquisition of children’s education (Aitken et al., 2019). An objective elimination mechanism does exist in higher education institutions, but the elimination criteria are the social strata of individual families (Althusser, 2019). Since the capital and habitus possessed by families from the dominant stratum potentially create symbolic violence, and the hidden exclusion from resources continues to aggravate the “involution” of education, equity becomes impossible to achieve (Gao et al., 2021).

According to Bourdieu and Passeron (1990), families with a high socioeconomic status (SES) provide extra educational resources for their children, leading to higher academic achievement in adolescents. Moreover, according to Coleman (1988), families with a high SES are often able to provide their children with a better living environment. As a result, their children have access to additional educational resources, and they do not need much parental involvement. Adolescents from low SES families benefit more from parental involvement than those from high SES families.

Based on the model of cultural mobility, Dimaggio (1982) argued that low SES levels encourage parents to invest in their children in order to compensate for other disadvantages. According to Liu et al. (2014), based on data from the Independent Freshman Admissions office at an elite university, adolescents from affluent families are more likely to succeed in the university admissions process. For adolescents in low SES families with less social capital, their parents’ involvement is more significant.

Due to the importance of access to higher education to future social stratification, relevant scholars in pedagogy and sociology have focused on “what factors lead to the differences in access to higher education for children of different families” (Mishra, 2020). Among the many factors affecting higher education access, family social status is considered the most essential structural element (Roksa and Kinsley, 2019; Liu and Morgan, 2020). The higher a family’s social status, the more likely its children are to receive higher education (Van Bavel et al., 2018). This conclusion conforms to Bourdieu’s theory of capital and has been confirmed by many other scholars (Romele, 2021).

The impact of family capital on children’s access to higher education (CAHE) is not applied directly. Instead, it is a long-term effect of certain specific factors. Although domestic scholars have analyzed in detail the family factors affecting CAHE in recent years, they have not yet explained the mechanism by which these factors influence CAHE, and authentic tests are rare. Therefore, based on existing research, this paper uses the theoretical perspective of family capital to reveal how contemporary Chinese families affect their CAHE.

In light of the fact that education has become increasingly important for class mobility, and that Chinese families face educational anxiety and social involution. This paper employs Bourdieu’s theory of capital—focusing on family cultural, social, and economic capital—to research the early-stage mechanism through which access to higher education is formed. While all three types of capital play a significant role in acquiring higher education, most studies tend to focus on just one type of capital. However, they have not yet explained the mechanism by which these factors influence CAHE, and authentic tests are rare. Therefore, based on existing research, this paper uses the theoretical concept of family capital to reveal how contemporary Chinese families affect their CAHE. As part of this study, we aim to gain a better understanding of the types of family capital that affect CAHE. This paper uses baseline data from the China Family Panel Studies (CFPS) from 2010 to 2020 to answer the following questions:

1. Does a significant difference exist in CAHE among children from families with different levels of family capital?
2. Is CAHE primarily influenced by the distribution of family capital?

## 2. Theoretical background and hypothesis

Due to its strong explanatory power, the concept of “capital” has been widely used across multiple research fields. French sociologist Bourdieu (2005) argued in “The Forms of Capital” that human capital takes diverse forms and can usually be divided into cultural, social, and economic capital. Accumulating any type of capital requires time and energy (Breinholt and Jæger, 2020). As a kind of accumulated labor, when exclusively possessed by actors or their aggregates, capital can provide social resources in a specific form. Cultural capital refers to a personal temperament that can be inherited and accumulated, such as interest preferences, knowledge literacy, and technical ability. These are usually represented by educational background and the possession of a diploma (Pokrovskaia et al., 2019). Social capital refers to the aggregate of actual or potential resources that individuals can obtain through an institutionalized social network, and it is the product of continuous effort, long-term operations, and the reciprocal communication of interest. This form of capital is usually characterized by professional status and number of family members (Ehsan et al., 2019; Shiell et al., 2020). Economic capital refers to explicit material wealth from which currency can be obtained by direct exchange. This capital includes operating, wage, and property income; government subsidies; and economic support from others, for example (Bauer and Zanjani, 2021).

Bourdieu’s distinction between these three types of capital has prompted scholars to examine how families with different cultural, social, and economic backgrounds take advantage of their existing capital to help their children attain a better education or higher social status, or to maintain their current status (Gilleard, 2020). Data analyzed from 14 universities in Jiangsu Province, China, showed that advantages related to family cultural capital are indirectly converted into educational opportunities, improving the likelihood that children will receive a higher education (Yao et al., 2015; Zhimin and Yao, 2015). In addition, Children from families with a high level of cultural capital have a better chance of studying at China’s top universities (Hu and Wu, 2021).

Du (2018) researched the influence of father’s occupational background on CAHE. The results showed that children with fathers in mid- and senior-level white-collar jobs are more likely to receive a higher education than children whose fathers have a lower occupational status. Wu et al. (2020) also noted that children from middle and upper class families enroll in higher education at a higher rate than those from lower class families.

Guo and Min (2006) established a relevant measurement model based on urban household survey data from the National Bureau of Statistics of China. Children whose families possess sufficient economic capital have a greater chance of receiving a higher education than children from poorer families. Kaye (2021) also confirmed that gaps in family income inevitably result in an inequality of access to higher education. However, Duan et al. (2018) noted that parental involvement, rather than economic conditions, is the primary source of family capital that contributes to CAHE.

Although all three types of capital are found to play a significant role in the acquisition of a higher education, most studies focus on a single type of capital. However, according to Bourdieu’s theory of multiple forms of capital, the study of the influence of family capital on CAHE cannot limit itself to one type. Instead, this question requires a comprehensive analysis of the action mechanism of cultural, social, and economic family capital (Li et al., 2020).

**Figure 1:**
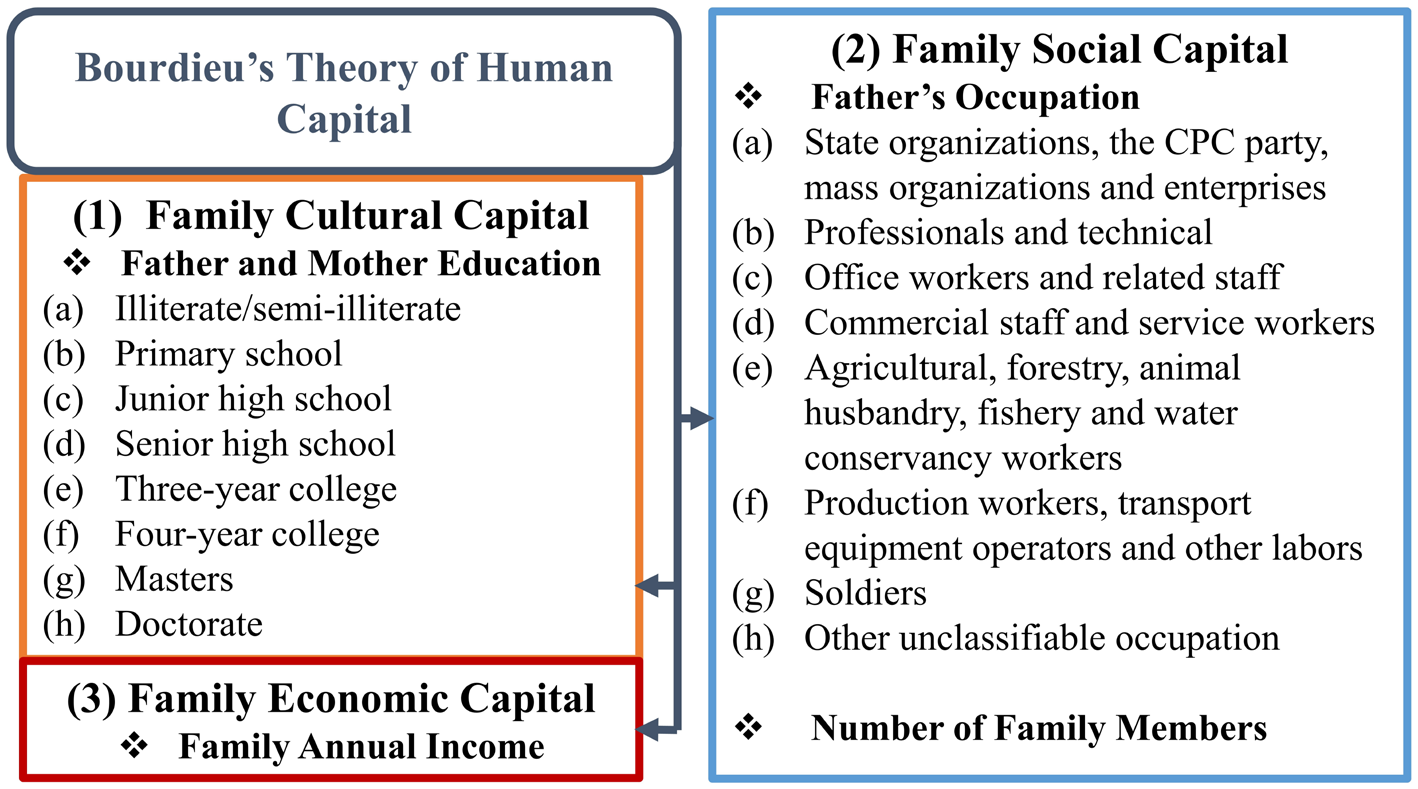
Based on Bourdieu’s theory of capital, indicators of data analysis.

This paper incorporates the three types of capital into one analytical framework. It compares the intensity and direction of their influence on CAHE for different families. Moreover, it considers the relationship between families’ strata and their children’s access to higher education. Following Bourdieu’s theory of human capital (Figure 1), this paper develops the following measurement indicators: (1) Father’s and mother’s highest academic qualification is selected as the measurement index of “family cultural capital,” and (2) father’s occupation and number of family members are selected as the measurement index of “family social capital.” Following the National Standard Occupational Classification of the People’s Republic of China, family size is determined by the number of family members with the same registered residence as the subject. Finally, (3) “family economic capital” is measured using the family’s total income over the past 12 months.

The literature review suggests that family capital has a significant effect on the access of children from different strata to higher education. Therefore, this paper puts forward the following three hypotheses:

*H_1_*: The amount of family cultural capital has a direct positive influence on CAHE.

*H_2_*: The amount of family social capital has a direct positive influence on CAHE.

*H_3_*: The amount of family economic capital has a direct positive influence on CAHE.

## 3. Methodology

### 3.1. Sample data and research process

Descriptive statistical methods were used in this study. All the data sets in this research come from the latest entries in the CFPS open database. By tracking and collecting microdata from three levels (individual, family, and community), the CFPS project surveys the economic and non-economic welfare of Chinese residents (CFPS, 2015). It tracks data in several research areas, such as educational achievements, economic activities, population migration, family relations and dynamics, and health through a multidisciplinary, large-scale social tracking survey at the national level. The CFPS project includes a broad sample and a rich volume of data. Its interviews began in 2010, with data from that year acting as the baseline. Since then, the data have been updated *via* interviews every 2 years. So far, the project has completed five tracking interviews, in 2012, 2014, 2016, 2018, and 2020, covering 25 provinces, municipalities, and autonomous regions. The target sample size is 16,000 families, and the survey’s object category is all the family members of a sample family.

The research process contains data collection and data analysis. In the process data collection, it focuses on respondents aged 18+ (as of 2020). The main variables include the following aspects: (1) basic information about the respondents, such as gender, academic qualifications, and registered residence; and (2) the respondents’ family background, namely parents’ education level, father’s occupation, number of family members, and annual family income. After discarding surveys with missing values, odd ratio, and other related variables, a total of 10,318 respondents were included in the research data set for use in the research hypothesis test. Zhimin and Yao (2015) used logistic regression analysis of survey data from 14 representative universities in Jiangsu Province is conducted to analyze the impact of family capital on the quantity and quality of higher education achieved by individuals. In line with previous studies, binary logistic regression model was used to analyze screened data to determine whether family capitals have a direct impact on CAHE.

### 3.2. Design variables

The dependent variable was set as “whether children can obtain higher education enrollment opportunities.” The independent variables were set as “father’s highest academic qualification (dummy variable),” “mother’s highest academic qualification (dummy variable),” “father’s occupation (dummy variable),” “number of family members (continuous variable),” and “family annual income (continuous variable).” The control variable was set as “gender of the sample (i.e., men and women as separate categories, with women as the base variable)” and “registered residence of the respondent (i.e., urban and rural as separate categories, with rural as the benchmark variable).”

The dependent variables were coded as follows: children have access to higher education = 0; children have no access to higher education = 1.

Parents’ highest academic qualification were coded as follows: illiterate/semi-literate = 1; primary school = 2; junior high school = 3; senior high school = 4; 3-year college = 5; 4-year college = 6.

Father’s occupation was coded as follows: the CPC Party, state organizations, mass organizations and enterprises = 1; professionals and technical = 2; employees of the office and related personnel = 3; workers in the commercial and service sectors = 4; forestry, agricultural, and animal husbandry workers and workers in the fields of fisheries and water conservation = 5; production workers, operators of transportation equipment and other laborers = 6; soldiers = 7.

### 3.3. Analysis model

**Figure 2:**
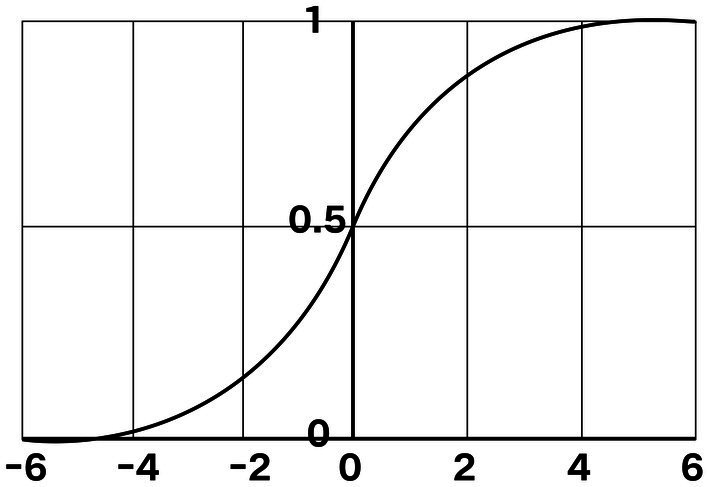
Logic distribution function curve.

To test the hypotheses, the logit model was adopted to investigate the influence of family capital on CAHE. Firstly, the logistic distribution function was used, expressed as *F*(x) = ex/(1 + ex), where the range of x is (−∞, +∞); the value range of *F*(x) is (0, 1) with a monotonically rising S-shaped curve (Figure 2).

Let the probability P of an event depend on multiple independent variables (X1, X2, …, XK); then P = eβ0 + β1X1 + β2X2 + … + βkXk + μ/(1 + eβ0 + β1X1 + β2X2 + … + βkXk + μ), where the range of P is (0, 1), and the range of β0 + β1X1 + β2X2 + … + βkXk + μ is (−∞, +∞). Then the relationship between the probability of the event and the variables fits the binary logistic regression model or the logarithmic dominance linear regression model.

That is, Ln[p/(1 – p)] = β0 + β1X1 + β2X2 + … + βkXk + μ, where β0 is a constant term, β1, β2, …, βk are the regression coefficients of k independent variables, and μ is the error term.

The following regression model was built according to the sample data in this paper:

L = Ln(opportunity)[p/(1 – p)] = β0 + β1 father’s edu + β2 mother’s edu + β3 father’s occupation + β4 number of family members + β5 family’s annual income + μ.

Let Y represent the explanatory variable, “whether children have access to higher education.” Because it is a binary variable, the study adopted the binary logistic regression model to analyze the ratio of the probability of “have access” and “have no access” in terms of higher education opportunities, with P being the probability of “have access” and 1 – P that of “have no access.” The ratio P/(1 – P) is called the chance ratio, and the logarithm L of the chance ratio is called the logarithmic unit.

## 4. Results

### 4.1. Descriptive statistical description

In the study, 10,318 pieces of valid data were selected. SPSS26.0 was used to classify the data based on urban versus rural area and gender (see Tables 1, 2). As shown in Table 1, among the selected participants from rural areas, 89.6% had completed a senior high school education or below, with 11.7% being illiterate/semi-illiterate. In contrast, 10.4% had a 3-year college degree or higher, 0.2% had a master’s degree, and none had a doctorate. This indicates that higher education has not been popularized among this population. While the academic qualifications of the rural population were generally low, the academic qualifications of the urban population were relatively high, with 29.7% having a 3-year college degree or higher, indicating the popularization of higher education among this population.

**Table 1:** Individual highest academic qualification and urban/rural classification variables based on data from the National Bureau of Statistics.

Items	Rural sample		Urban sample			F	%	F	%	Total
Illiterate/semi-literate	651	11.7	223	4.7	874
Primary school	1,124	20.2	469	9.8	1,593
Junior high school	2,132	38.4	1,473	30.9	3,605
Senior high school	1,073	19.3	1,185	24.9	2,258
3-year college	342	6.2	673	14.1	1,015
4-year college	222	4.0	679	14.3	901
Master’s degree	10	0.2	56	1.2	66
Doctoral degree	1	0.0	5	0.1	6
Total	5,555	100.0	4,763	100.0	10,318

**Table 2:** Individual highest academic qualification and personal gender.

Items	Female sample		Male sample			F	%	F	%	Total
Illiterate/semi-literate	509	11.5	365	6.2	874
Primary school	599	13.6	994	16.9	1,593
Junior high school	1,441	32.6	2,164	36.7	3,605
Senior high school	941	21.3	1,317	22.3	2,258
3-year college	467	10.6	548	9.3	1,015
4-year college	428	9.7	473	8.0	901
Master’s degree	33	0.7	33	0.6	66
Doctoral degree	1	0.0	5	0.1	6
Total	4,419	100.0	5,899	100.0	10,318

As shown in Table 2, among the selected female respondents, 79.0% have a senior high school education or below, with as many as 11.5% being illiterate/semi-illiterate. Meanwhile, 21.0% have college-level qualifications or above. In contrast, among the male respondents, 82.1% have a senior high school education or below, with only 6.2% being illiterate/semi-illiterate. This was 5.3% lower than the female respondents. Among the male respondents, 18.0% have 3-year college degree or above, which was 3.0% lower than the female respondents. As a whole, the female population shows a more obvious “bipolar” differentiation in terms of education level.

### 4.2. Evaluation of the structural model

According to the regression model, L = Ln(opportunity)[p/(1 – p)] = β0 + β1 father’s edu + β2 mother’s edu + β3 father’s occupation + β4 number of family members + β5 family’s annual income + μ. By regressing the data using the binary logistic command of the SPSS26.0 software, the sample processing results were obtained (see Table 3).

**Table 3:** Case processing summary.

Unweighted cases^a^		*N*	Percentage
Selected cases	Included in analysis	10,318	100.0
	Missing cases	0	0.0
	Total	10,318	100.0
Unselected cases		0	0.0
Total		10,318	100.0

^a^Constant is included in the Model & represents unweighted cases.

Table 4 presents the misjudgment matrix of the model. In this study, among the 8,330 people without access to higher education in the actual samples, 8,093 were correctly identified by the model and 237 were wrongly identified, for a correct rate of 97.2%. Among the 1988 people with access to higher education in the actual samples, 393 were correctly identified by the model and 1,595 were wrongly identified, for a correct rate of 19.8%. The field “the cut value is 0.500” in the footnote under the diagram indicates that if the prediction probability value was greater than the critical value of 0.5 (i.e., 50%), the correct prediction rate of variable classification was considered to be 100%. Otherwise, the correct prediction rate of variable classification was considered to be 0. Therefore, the correct prediction rate of the above model was 82.2%, and the result was within the acceptable scope (i.e., greater than 50%).

**Table 4:** Final prediction classification table (Forward: LR).

	Observed		Predicted		
			Individual higher education opportunities		Percentage correct
			0	1	
Step 5	Individual higher education opportunities	0	8,093	237	97.2
		1	1,595	393	19.8
	Overall percentage				82.2

^a^Constant is included in the model. ^b^The cut value is 0.500.

Table 5 presents the significance test results of the model. Step statistics refers to the likelihood ratio test results of each step compared with the previous step. Block refers to the likelihood ratio test results of Block _n_ compared with Block_n – 1_. Model refers to the likelihood ratio test results of the model after the variables in the previous model and the current model change. When Method = Forward: LR was selected, the three statistical results and the hypothesis test results were completely consistent. The above statistical results showed that the regression model passed the significance test.

**Table 5:** Omnibus tests of model coefficients (Forward: LR).

		Chi-square	df	Sig.
Step 1	Step	720.900	6	0.000
	Block	720.900	6	0.000
	Model	720.900	6	0.000
Step 2	Step	262.768	7	0.000
	Block	983.667	13	0.000
	Model	983.667	13	0.000
Step 3	Step	231.784	1	0.000
	Block	1215.451	14	0.000
	Model	1215.451	14	0.000
Step 4	Step	211.224	1	0.000
	Block	1426.675	15	0.000
	Model	1426.675	15	0.000
Step 5	Step	70.732	6	0.000
	Block	1497.407	21	0.000
	Model	1497.407	21	0.000

^a^Variable (s) entered in step 1: Mother’s education; ^b^Variable (s) entered in step 2: Father’s occupation; ^c^Variable (s) entered in step 3: Number of family members; ^c^Variable (s) entered in step 3: Number of family members; ^d^Variable (s) entered in step 4: Family’s annual income; ^e^Variable (s) entered in step 5: Father’s education.

When the significance value (Sig) is less than the cut value (i.e., 0.05), the null hypothesis is rejected. In this case, no significant difference was found between the observed value of the dependent variable and the predicted value of the model, so there was a significant difference between the predicted value and the observed value of the model. Otherwise, the null hypothesis could not be rejected, and the estimation of the model fit the data at an acceptable level. According to the goodness of fit test results in Table 6, in Step 5, the X2 value corresponding to the Hosmer–Lemeshow Test statistics of the model was 9.775, the degree of freedom was 8, and the Sig value was 0.281; that is, Sig > 0.05, and the difference was not significant. Therefore, the hypothesis that the predicted value would be consistent with the observed value could not be rejected. This indicated that the model had sound goodness of fit. Because of the difference between the predicted value of the model and the observed value, the hypothesis was accepted. There was a significant correlation between CAHE and family capital.

**Table 6:** Hosmer and Lemeshow test (Forward: LR).

Step	Chi-square	df	Sig.
1	0.000	2	1.000
2	3.219	5	0.666
3	26.928	8	0.001
4	10.598	8	0.226
5	9.775	8	0.281

Table 7 presents the significance test results of the model parameters. The variables passing the Forward: LR test were father’s highest academic qualification, mother’s highest academic qualification, father’s occupation, number of family members, and family’s annual income. According to the Sig value of the significance of the regression coefficient, father’s highest academic qualification (Sig < 0.001), mother’s highest academic qualification (Sig < 0.001), father’s occupation (Sig < 0.001), number of family members (Sig < 0.001), and family’s annual income (Sig < 0.001) were all found to have a significant influence on CAHE.

**Table 7:** Variables in the equation (Forward: LR).

		B	S.E	Wald	df	Sig.	Exp (B)
Step 5	Father’s education	^***^		69.820	6	0.000	
	Illiterate/semi-literate	0.346^***^	0.092	13.992	1	0.000	1.413
	Primary school	0.557^***^	0.091	37.286	1	0.000	1.746
	Junior high school	0.833^***^	0.107	61.027	1	0.000	2.300
	Senior high school	0.855^***^	0.184	21.705	1	0.000	2.352
	3-year college	0.504	0.261	3.746	1	0.053	1.656
	4-year college	0.429	1.510	0.081	1	0.776	1.536
	Mother’s education	^***^		145.972	6	0.000	
	Illiterate/semi-literate	0.320^***^	0.074	18.623	1	0.000	1.377
	Primary school	0.699^***^	0.076	84.961	1	0.000	2.013
	Junior high school	1.077^***^	0.105	104.567	1	0.000	2.935
	Senior high school	0.805^***^	0.197	16.696	1	0.000	2.236
	3-year college	0.143	0.333	0.186	1	0.667	0.866
	4-year college	22.033	40192.969	0.000	1	1.000	3,706,706,030
	Father’s occupation	^***^		110.755	7	0.000	
	State organizations, the CPC Party, mass organizations and enterprises	0.237	0.215	1.223	1	0.269	0.789
	Professionals and technical	0.002	0.209	0.000	1	0.991	0.998
	Office workers and related staff	−0.430^*^	0.202	4.518	1	0.034	0.651
	Commercial staff and Service workers	−0.970^***^	0.181	28.743	1	0.000	0.379
	Agricultural, forestry, animal husbandry, fishery and water conservancy workers	−0.426^*^	0.183	5.422	1	0.020	0.653
	Production workers, transport equipment operators and other labors	0.864	1.204	0.515	1	0.473	2.374
	Soldiers	−0.609^**^	0.189	10.327	1	0.001	0.544
	Number of family members	−0.239^***^	0.016	234.940	1	0.000	0.787
	Family’s annual income	0.000^***^	0.000	180.028	1	0.000	1.000
	Constant	−0.895	0.207	18.752	1	0.000	0.409

(a)^*^indicates that sig < 0.05; ^**^indicates that sig < 0.01; ^***^indicates that sig < 0.001.

Firstly, parents’ academic qualification has a significant positive influence on CAHE. The higher the parental academic qualification, the greater the probability of CAHE. Therefore, hypothesis H1 is accepted. As shown in Table 7, both parents’ highest academic qualification (illiterate/semi-illiterate, primary school, junior high school, and senior high school) significantly influenced the explained variables at a significance level of 1%. Notably, the mother’s academic qualification had a greater influence on CAHE than the father’s academic qualification, if viewed from the absolute value of regression coefficient B.

Secondly, when the father’s occupation was an official of a state organization, head of a mass organization or enterprise, professional or technical worker, production worker, or transport equipment operator, the regression coefficient B was positive. This is conducive to CAHE opportunities. In contrast, when the father’s occupation was office worker; commercial staff or service worker; agricultural, forestry, animal husbandry, or fishery and water conservancy worker; or soldier, the regression coefficient B was negative. This is not conducive to CAHE opportunities. Among these, father’s occupation (commercial staff and service worker) was significant at a significance level of 1%, father’s occupation (soldier) was significant at a significance level of 1%, and father’s occupation (office worker, agricultural, forestry, animal husbandry, or fishery and water conservancy worker) was significant at a significance level of 5%. Consequently, hypothesis H_2_ is accepted. Notably, as the father’s occupational status decreased, the probability of CAHE did not decrease accordingly. When the father’s occupation was production worker or transport equipment operator, the probability of CAHE rebounded significantly, which may be directly correlated to the Chinese population’s social and industrial structure.

Thirdly, number of family members had a significant influence on the explained variables at a significance level of 1%. At the same time, the number of family members was negatively correlated with CAHE. That is, as the number of family members increased, the probability of CAHE decreased accordingly.

Finally, annual family income had a significant positive influence on the explained variables at a significance level of 1%. Thus, hypothesis H_3_ has been accepted. This shows that when other conditions remain unchanged, the higher the annual family income, the greater the probability of CAHE.

## 5. Discussion

### 5.1. Influence of family cultural capital on children’s access to higher education

As a result of the empirical findings, it is confirmed that the possession of family cultural capital has a direct positive influence on CAHE, which supports the research hypothesis. Based on the empirical evidence, research hypothesis H_1_ has been accepted. This study shows that the possession of family cultural capital has a direct positive influence on CAHE. This may be directly related to intellectual factors, personal effort, supply of learning resources, or parental guidance, for example. Lissitsa and Chachashvili-Bolotin (2021) found that variations in individual effort, learning resources, and parental guidance stem largely from different levels of family cultural capital. Limited cultural capital negatively impacts children’s preparation and judgment when aiming toward higher education opportunities. These conclusions mirror the findings of another study that parents who are rich in cultural capital have a relatively positive attitude toward their children entering higher education. They consciously increase their educational investment, interact more with their children, and potentially encourage their children’s cultural literacy, interests, tastes, and ideas with a benign family cultural atmosphere (Cheng and Kaplowitz, 2016; Hu and Yin, 2021). Under such parental influence, these children often have a strong internal motivation to strive for higher education opportunities. They hope to develop their professional abilities through higher education and realize their own potential values. In contrast, families with weak cultural capital have comparatively ambivalent views on higher education. Most parents do not have a higher education background and are unlikely to recognize its value for their children’s future and personal growth. This parental ambivalence continues to restrict children’s understanding of higher education through inter-generation transmission (Hao and Pilz, 2021). Hu and Wu (2021) argued that families with more economic capital do not always have more cultural capital. They maintained that the key factor affecting family cultural capital is the parents’ academic qualifications. For example, the Chinese College Entrance Examination is designed to screen elite students. Parents with high academic qualifications have accumulated more cultural capital, so they can give their children an early competitive advantage. This inter-generation transmission is gradually converted into an exam score advantage.

Interestingly, this study finds that the influence of family capital on access to higher education is significantly greater for females than for males. Similarly, Si (2022) found that if a female comes from a family with better economic conditions, her family conditions have a greater positive influence on her access to education opportunities. In addition, compared to men, women’s access to higher education is more limited by family economic background (Guo et al., 2018).

The current study also found that mothers have a significantly greater influence than fathers on their CAHE, which may result from family tradition and the social division of labor. As Zhang et al. (2020) explained, mothers pay more attention than fathers to their children’s early social development and assume the most responsibility for their children’s education. In particular, the cultural capital investment of mothers from middle-stratum families is advantageous for their children’s educational achievements. This finding is similar to Hou et al.’s (2008) conclusion that mothers’ academic qualifications have significantly more influence on their CAHE than fathers’ academic qualifications.

### 5.2. Influence of family social capital on children’s access to higher education

A significant influence of family social capital on CAHE is confirmed by the empirical findings, thus supporting the research hypothesis. As previous studies have shown, parents with close social relations and shared values across a social network can rely on these relationships to receive support from others. Moreover, they can exploit the social capital in their relationship network to increase their children’s competitive advantage in education (Han, 2019). In modern eastern China, the elite screening system represented by the College Entrance Exam is the principal gateway to obtaining higher education opportunities. However, the competition for access to higher education opportunities begins long before the College Entrance Examination. This competition is often reflected in the family’s choice of educational resources for their children during the stages of preschool, primary school, junior high school, and senior high school. Currently, the distribution of educational resources in China indicates that eastern China is better resourced than central and western China, urban areas have more resources than rural areas, and key schools are better resourced than ordinary schools (Li and Qiu, 2018). In contrast, families with weak social capital usually have few powerful social relationships and a single source of information on higher education. This significantly limits parents’ expectations that their children will obtain higher education (Chen and Liu, 2021).

The regression analysis also found that children in larger families are less likely to access higher education. This may be directly related to parental values. Hoffmann et al. (2020) demonstrated that the number, gender, and especially the age of children in families with less social capital directly affect how much the parents invest in educational resources. However, another study (Zhu, 2019) suggested that the development of national modernization has weakened the influence of hereditary factors such as sex and race on children’s access to education. At the same time, the influence of individual effort has strengthened.

### 5.3. Influence of family economic capital on children’s access to higher education

According to the results, the level of family economic capital has a positive influence on CAHE, which confirms the research hypothesis. Since its reform and opening up, China’s market economy has developed vigorously. Under the production principle of giving priority to efficiency and consideration to fairness, the economic gaps between different strata and regions have expanded accordingly. The resulting differences in family economic capital have directly affected the continuity of children’s access to education. As another study found, the influence of family economic capital on CAHE is gradually increasing with the marketization of China’s higher education. Children from families with abundant economic capital can obtain more high-quality educational resources through after-school instruction and private tutors. If they fail their entrance examinations, they may still enroll in foreign universities. However, children from families with relatively weak family economic capital lack the means to access high-quality educational resources. Moreover, the expense of higher education potentially suppresses their academic expectations. Their material poverty and reduced expectations push them to consciously give up on opportunities for further study (Wu et al., 2020).

A previous study analyzed the average cost for undergraduate students attending certain colleges and universities in Beijing and found that students must bear a minimum personal cost of 11,596 RMB every year (Shen and Luo, 2021). The cost of higher education for economically disadvantaged families has decreased in recent years due to the improvement of the national scholarship and student loan system. However, living expenses—including clothing, food, housing, and transportation—that families must bear for their children during school remain higher than 10,000 RMB (Cai et al., 2019; Yang, 2022). Moreover, such an expenditure cannot be repaid in the short term. At the same time, since the expansion of college enrolment, the employment situation has become more competitive, while education costs have not decreased. Children from economically disadvantaged strata—especially those from rural families—often choose to terminate their studies at a particular educational transition stage, making the pragmatic judgment to enter employment as soon as possible for the economic benefit of their families (Mok and Marginson, 2021).

The results presented in this paper show that some children from the blue-collar stratum also have a comparative advantage in obtaining higher education opportunities. Mishra (2020) argued that individuals whose parents have weak social relationships may also be able to obtain higher educational returns. Due to the insecure environment of these disadvantaged strata, their members value educational opportunities and are eager to improve their social status through higher education. Furthermore, families with scarce social capital may concentrate on family capital to enhance their children’s educational competitiveness and help them pass the standardized entrance examination (Mishra, 2020). However, most families from disadvantaged strata are likely to consciously avoid or abandon this high-risk strategy.

### 5.4. Implications

This study concludes that family capital is an important factor affecting the stratification of CAHE. A family’s economic capital has a direct positive influence on CAHE. However, family cultural capital has a greater direct positive influence on CAHE. Families with low incomes are more likely to have parents with low education levels. This limits the educational resources they can offer their children. When parents in low-income families have greater cultural capital (e.g., a higher education), they can help their children be more competitive. Due to their weak risk tolerance, low-income families can benefit from government investment in economically disadvantageous regions. The government should improve its gradient compensation mechanism to reduce regional differences in the allocation of educational resources. In particular, the parents in low-income families tend to be less educated. These parents should cooperate with schools and communities to take professional courses in assisted learning, emotional counseling, decision-making, and voluntary service. Schools and parents can also learn from the Parent Teacher Association (PTA) model in the United States to increase parents’ involvement in their children’s education.

### 5.5. Limitations

This study did not include the entire sample from the CFPS (2010–2020) database, thus reducing the representativeness of the findings. Additionally, the authors lack a comprehensive understanding of alternative theories in demography, politics, and sociology, which would enable them to provide a more comprehensive assessment of equity in higher education. Future studies will build a stronger theoretical framework by using a broader sample. A more accurate mathematical model will also be developed. This will allow us to investigate how family capital influences children’s education acquisition at different levels and types of colleges and universities.

## 6. Conclusion

This study concludes that family capital is an important factor affecting the stratification of CAHE. Compared with family economic capital, family cultural capital has a more significant influence, benefiting children from elite strata. In addition, the influence of family capital on access to higher education is significantly greater for females than for males. The current study also found that mothers’ education has a significantly greater influence than fathers’ education on CAHE, which may result from family tradition and the social division of labor in China. Although family social capital influences access to higher education, no clear difference was found between strata. China’s political, economic, and social environment gives some children from the blue-collar stratum a comparative advantage. The regression analysis shows that children’s access to higher education does not decrease with the father’s lower occupational status. It also found that children from larger families are less likely to access higher education. Moreover, the children of production workers and transport equipment operators have a significantly higher probability of accessing higher education. These results demonstrate that not all forms of family capital create strata differences in terms of access to higher education. In poor families, family cultural capital has a greater influence on improving children’s competitive advantage in accessing higher education opportunities. When parents in low-income families have more cultural capital (e.g., higher education), they can help their children be more competitive.

## Data availability statement

The original contributions presented in the study are included in the article/supplementary material, further inquiries can be directed to the corresponding author.

## Ethics statement

Ethical review and approval was not required for the study on human participants in accordance with the local legislation and institutional requirements. Written informed consent for participation was not required for this study in accordance with the national legislation and the institutional requirements.

## Author contributions

CL and QT presented the main idea and wrote the first draft of the manuscript. SA and CL interpreted the data and drafted the manuscript. PW reviewed the manuscript. SA and LT revised the manuscript based on the critical comments and finished the final submission. All authors contributed to the article and approved the submitted version.

## Funding

The authors disclosed receipt of the following financial support for the research, authorship, and/or publication of this article: This work was partially supported by the Chinese Society for Technical and Vocational Education and the China Institute of Vocational and Technical Education for a New Era in 2022 (grant no. SZ22B34).

## Conflict of interest

The authors declare that the research was conducted in the absence of any commercial or financial relationships that could be construed as a potential conflict of interest.

## Publisher’s note

All claims expressed in this article are solely those of the authors and do not necessarily represent those of their affiliated organizations, or those of the publisher, the editors and the reviewers. Any product that may be evaluated in this article, or claim that may be made by its manufacturer, is not guaranteed or endorsed by the publisher.
